# Fast Response and High Sensitivity ZnO/glass Surface Acoustic Wave Humidity Sensors Using Graphene Oxide Sensing Layer

**DOI:** 10.1038/srep07206

**Published:** 2014-11-26

**Authors:** Weipeng Xuan, Mei He, Nan Meng, Xingli He, Wenbo Wang, Jinkai Chen, Tianjin Shi, Tawfique Hasan, Zhen Xu, Yang Xu, J. K. Luo

**Affiliations:** 1 Dept. of Info. Sci. & Electric. Eng., Zhejiang University and Cyrus Tang Center for Sensor Materials and Applications, 38 Zheda Road, Hangzhou 310027, P.R. China; 2 Cambridge Graphene Centre, University of Cambridge, Cambridge CB3 0FA, United Kingdom; 3 MOE Key Lab. of Macromolecul. Synth. & Funct., Dept. of Polym. Sci. & Eng., Zhejiang University, 38 Zheda Road, Hangzhou 310027, P. R. China; 4 Inst. of Renew. Energ. & Environ. Technol., University of Bolton, Deane Road, Bolton BL3 5AB, United Kingdom

## Abstract

We report ZnO/glass surface acoustic wave (SAW) humidity sensors with high sensitivity and fast response using graphene oxide sensing layer. The frequency shift of the sensors is exponentially correlated to the humidity change, induced mainly by mass loading effect rather than the complex impedance change of the sensing layer. The SAW sensors show high sensitivity at a broad humidity range from 0.5%RH to 85%RH with < 1 sec rise time. The simple design and excellent stability of our GO-based SAW humidity sensors, complemented with full humidity range measurement, highlights their potential in a wide range of applications.

Measurement and control of environment humidity are of great importance in industrial processes, agricultural productions, and research experiments^1^,^2^,^3^. Various types of humidity sensors have been developed based on the mechanisms such as capacitance^4^, resistance^2^,^5^,^6^,^7^ *etc*. However, the ever-fast progressing science and technology require humidity sensors with better sensitivity, wider sensing range, faster response, shorter recovery time, and lower cost than what is now commercially available^8^. Considerable efforts have thus been focused on the development of highly sensitive materials and novel device structures to address these issues. Because of very high surface to volume ratio and special physical/chemical properties^9^,^10^, use of nanomaterials such as ZnO nanorods^11^, carbon nanotubes^12^, electrospun nanofibers^13^, metal oxide nanowires^14^,^15^ *etc* in humidity sensors has resulted in many exciting progresses over the recent years. However, the development of novel sensitive materials and high performance humidity sensors, especially for applications requiring fast, low humidity level detection deserve more investigation and research.

Functionalized graphene exhibits unique properties in chemical and biological sensing^16^,^17^,^18^,^19^,^20^. Graphene oxide (GO), a derivative of graphene with oxygen functional groups, has attracted much attention owing to its considerable advantages for detecting gas^21^ and biological substances^22^. GO also shows excellent humidity sensing capability because of its large surface area to volume ratio and hydrophilic functional groups^4^,^23^.

Surface acoustic wave (SAW) devices are one of the building blocks for electronics^24^, microsensors^25^, and microsystems^26^. SAW based humidity sensors have undergone many investigations owing to their high sensitivity, small size, and ability to be interfaced with passive wireless systems, *etc*^27^,^28^. A flexible ZnO thin film SAW humidity sensor without additional sensing layer was proposed by He *et al*^29^, with a sensitivity of ~3.47 kHz/%RH at 85%RH. Lin *et al* reported a SAW humidity sensor based on electrospun polyaniline/poly(vinyl butyral) nanofibers sensitive layer with a sensitivity of ~75 kHz/%RH from 20 to 90%RH^13^. Li *et al* investigated SAW humidity sensors with silver nanoparticles as the sensing layer and obtained a sensitivity of over 10.6 kHz/%RH^30^. Balashov *et al* demonstrated humidity sensing characteristics of SAW sensors with atomizing GO sensitive layer with a sensitivity of 1.54 kHz/%RH^31^. Whitehead *et al* and Ciplys *et al* investigated the mechanisms of SAW sensors with a graphene layer, and pointed out that the mass loading and viscoelastic effects are responsible for the increased attenuation observed^19^,^20^. However graphene is hydrophobic, and is not particularly suitable as a high sensitive sensing layer for humidity sensors. Although the majority of the humidity sensors reported to date show good performance in one or two parameters (such as sensitivity or response speed), they still lack overall performance involving all parameters, a key requirement for applicability in real-world usage. In this paper, we report SAW humidity sensors based on ZnO piezoelectric thin film on low cost glass substrate with GO as the performance-enhancement sensing layer. The humidity sensors exhibit high sensitivity from low (0.5%RH) to high (85%RH) humidity range with very fast response (rise and fall time of <1 sec and ~19 sec, respectively), demonstrating its great potential for application in broad humidity range with high-speed detection.

## Results

### Graphene oxide films and humidity sensors

We prepared GO nanoflakes from natural graphite crystals using methods reported elsewhere^32^. The GO flakes have good solubility in water because of their oxygen-containing functional groups. The as-prepared GO dispersion (4.6 mg/ml) was diluted from a deionized (DI) water solution to the concentrations of 0.046, 0.184 and 0.460 mg/ml, respectively. The diluted GO dispersions were then homogenized in an ultrasonic bath sonicator (power 360 W) for 15 min. These different GO dispersions were then used to form the sensing layers with different thicknesses.

**Figure 1:**
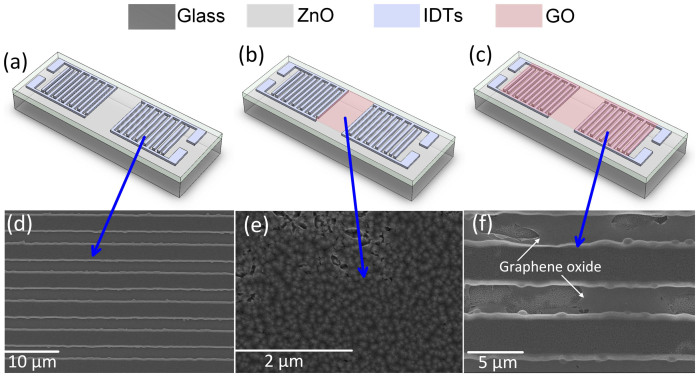
Schematics of the SAW devices (a) with clean surface, covered by a GO layer (b) in the middle, and (c) the whole area. SEM images of the IDTs (d) with clean surface, coated with a GO (e) in the middle, and (f) in the whole area.

GO dispersions allow deposition on target substrates by various methods such as drop-, spin-, spray-, and dip-coating^23^,^33^. In this study, GO layers were deposited by either spin-coating or drop-casting on the device surfaces. For spin-coated samples, thin GO layers were deposited on the surface of the devices at 1500 rpm for 20 sec. While for the drop-cast ones, we deposited GO on the target surface area using a micro-syringe. After the deposition of GO, the devices were kept at room temperature to allow slow evaporation of water. Figure 1a,b,c show the schematics of the SAW sensors used for the measurements, while Figure 1d,e,f are the SEM images of the SAW devices with a clean surface, a drop-cast GO layer in between the aluminum (Al) interdigitated transducers (IDTs) and covering the whole surface, respectively. The areas with partial GO coverage were deliberately chosen for the SEM images to clearly illustrate where the GO is, as shown in Figure 1e, f. The thickness of the drop-cast GO layer is 70-300 nm as measured by profilometer, depending on the concentration of GO used for the deposition. Information on GO layer thickness is presented in Figure S1 in the Supplementary Information (SI).

**Figure 2:**
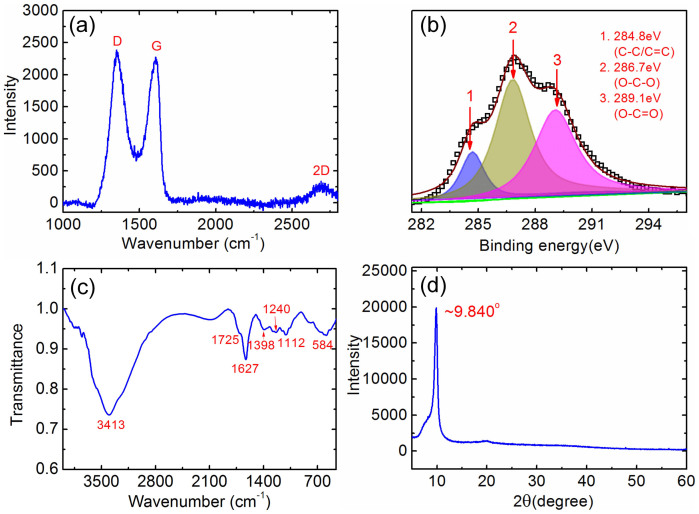
Characterization of GO layer used in SAW based humidity sensor. (a) Typical Raman spectrum, and the excitation laser wavelength used is 532 nm. (b) XPS spectrum. (c) FT-IR spectrum. (d) XRD pattern of the GO layers used in this work.

The typical Raman spectrum of the GO layers is shown in Figure 2a. Two most prominent peaks appear at 1590 cm^−1^ and 1350 cm^−1^ which are known as the G and D peaks, respectively. The G peak corresponds to the E_2g_ phonon at the Brillouin zone center. In pristine graphene^34^, it appears at 1580 cm^−1^. The presence of double-bonds (C = C) in our GO sample causes the blue-shift of the G band^35^ to ~1590 cm^−1^. The D peak originates from the breathing modes of six-atom rings and requires a defect for its activation. For the case of GO, this primarily comes from lattice defective states (C = O, C-H, C-O, C-O-C, C-OH). Also, for the case of defected graphene samples like GO, G peak broadening is commonly observed as a result of the activation of q≠0 phonons^36^. This was observed in our GO samples, with Full Width Half Maximum of G, FWHM(G)~80 cm^−1^. The high I_D_/I_G_ ratio (~1.1) indicates large defect density in our GO sample. Considering FWHM(G) and I_D_/I_G_ in our sample, we use I(D)/I(G) = C′(λ)L_a_^2^ to find the cluster size, L_a_, with C′ = 0.55 nm^−2^ 37. This gives us L_a_ ~ 1.4 nm. Figure 2b shows a typical XPS C1s spectrum of the GO layer obtained with MgKa X-ray source (1253.6 eV). This spectrum is very broad, and can be deconvoluted into three components, located at 284.8 eV, 286.7 eV and 289.1 eV, respectively. These represent the binding energies of C-C SP^2^, O-C-O and O-C = O bonds, respectively. These oxygen-related chemical bonds increase the hydrophilicity of the GO layers, thus enhancing the humidity sensitivity of the SAW devices^38^.

Figure 2c shows FT-IR spectrum obtained from our GO samples. A strong and broad valley at 3413 cm^−1^ is attributed to the -OH groups in stretching vibrations. This peak implies the good hydrophilicity of the GO samples used for our humidity sensing. Other absorption peaks at 1725 cm^−1^ originates from the C-O stretching vibrations of carbonyl and carboxylic groups, at 1627 cm^−1^ from the C-H, at 1398 cm^−1^ from the C-OH, at 1240 cm^−1^ from the C-O-C and at 1112 cm^−1^ of C-O stretching. These are all consistent with the results of the XPS spectrum. The presence of such carboxyl and hydroxyl functional groups in GO enhances the water adsorption ability of the sensing layer for humidity sensing.

XRD pattern of the GO layers used in our experiments is shown in Figure 2d. The peak at 2θ = 9.84° corresponds to an interlayer spacing (d-spacing *λ* = 2*d* sin(*θ*)) of about 9.0 Å, which is much larger than that of bulk graphite crystal (3.3 Å, 2θ = 26.3°)^4^, indicating the nature of the layer is very different from graphite. The large interlayer spacing of GO reflects the existence of rich oxygen-containing functional groups in the GO layers^39^. In addition, the single dominant peak indicates the uniformity of the GO samples.

**Figure 3:**
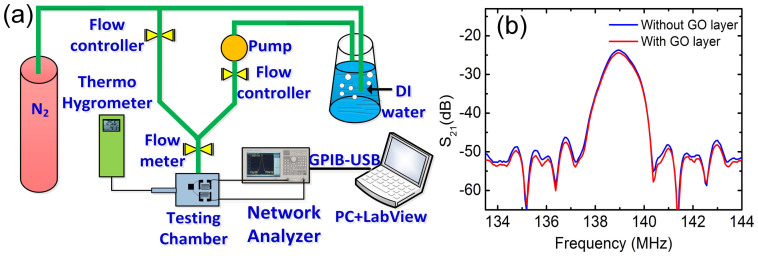
(a) Diagram of the experimental setup for the humidity sensing. (b) Typical transmission spectrum of the SAW sensors before and after GO coating.

Figure 3a presents the experimental setup schematic. The sensors were placed in a hermetic box with two through-holes for humidity N_2_ gas to pass through for sensing. The relative humidity in the box was controlled by changing the flow ratio of dry N_2_ to wet N_2_ into the box, while keeping the total flow rate at 500 sccm. All the experiments were conducted at 25 ± 2°C.

SAW sensors with wavelengths of *λ* = 12 and 20 μm and resonant frequencies at ~225 MHz and ~140 MHz, respectively, were fabricated. They were coated with various GO layers for the humidity sensing experiments. Table 1 is the summary of the samples with different device structures or GO layers. For ease of comparison, we classify the sensors with similar parameters into group A, B, and C as summarized in Table 2. Figure 3b shows a typical transmission spectrum of a SAW sensor before and after coating with GO at ~50%RH. After deposition of the GO layer, both the central (or resonant) frequency and signal amplitude of the devices decrease slightly as a result of the mass (GO layer) loading effect^40^, allowing sensing of the humidity change. The relatively small shift of the resonant frequency, ~425 Hz also indicates the amount of GO mass introduced is small, such that it would not change the device characteristics significantly. We found that thick GO layer can render the device inoperable with drastically deteriorated resonant spectrum and transmission (see Figure S2 in SI) as thick non-piezoelectric GO layer acts as an effective damping layer, absorbing large proportion of acoustic energy. We therefore limited the GO thickness below 300 nm for this work.

### Sensor response to humidity

**Figure 4:**
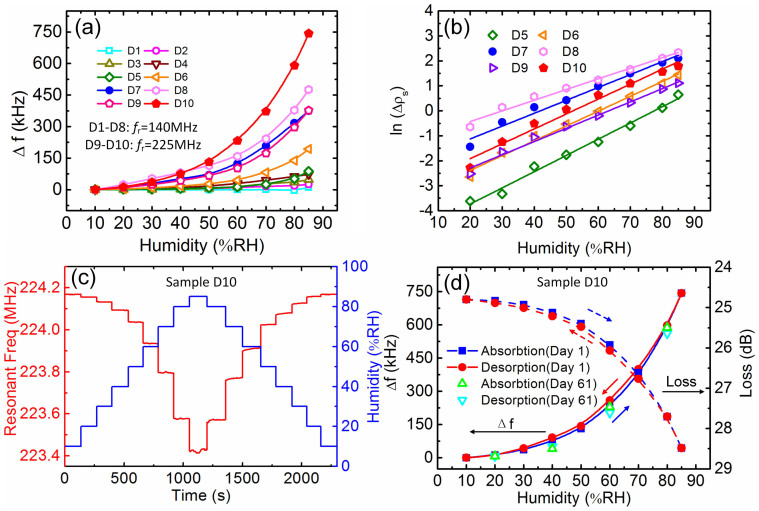
(a) The resonant frequency shift as a function of relative humidity for sensors with different surface treatments. Devices from D1 to D8 are sorted to Group A with *f_r_*~140 MHz. (b) The adsorbed surface mass density as an exponential function of relative humidity is found with excellent linearity (*R^2^* of samples D5, D6, D7, D8, D9, and D10, are 0.9881, 0.9889, 0.9773, 0.9842, 0.9890 and 0.9763, respectively, *R^2^* is the correlation coefficient). (c) Frequency response of the SAW sensor (D10) to RH change from 10%RH to 85%RH, then return to 10%RH. (d) Frequency and insertion loss shift vs. humidity for sample D10, showing good stability and little hysteresis even after being exposed to a large range of fast humidity change from Day 1 to Day 61.

Moisture can be easily absorbed by the GO layer as it is a hydrophilic material (depending on the level of oxidation/functionalization)^41^,^42^. GO also contains oxygen epoxide and hydroxyl groups^43^,^44^,^45^, which can increase its hydrophilicity^46^, thus enhancing the humidity sensitivity of the sensors by adsorbing or desorbing more water molecules. This can lead to a change in the mass and conductivity of the sensing layer^4^,^23^,^27^, shifting the resonant frequency and other transmission parameters of the SAW sensor. Note that the change in viscoelastic property of a coating layer upon absorption of water moisture may also increase the insertion loss and induce frequency shift, and is believed to be one of the causes responsible for the graphene coated SAW humidity sensors^19^,^20^. However, because of high shear modulus of the interlocked GO flakes^47^, we do not foresee this to be a major contributor to the sensing mechanism in our devices. Figure 4a is the summary of the resonant frequency change vs. humidity for sensor D1-D10 with various resonant frequencies and GO layers. For a fixed frequency of *f_r_*~140 MHz in Group A, sample D8 with a GO thickness of 200–300 nm over the whole surface exhibits the most significant frequency shift, *i.e.* the highest sensitivity, followed by the sensors with thinner or smaller area GO layers. Similarly, for the devices with a frequency of *f_r_*~225 MHz, D10 with a thicker GO layer has a larger frequency shift than that of D9 with a thinner GO layer.

The resonant frequency change of the sensors increases with the rise of humidity with a nonlinear characteristic for all the sensors tested. As mentioned above, the change in mass^40^ and conductivity^48^ of the sensitive materials or piezoelectric surface layer are two main variables that may induce resonant frequency shift of the SAW sensors. In this work, we demonstrate that the frequency shift is primarily caused by the mass loading effect of the adsorbed water, to be discussed later in detail. In general, the frequency change, *Δf*, induced by a loaded mass, *Δm*, can be expressed as^7^

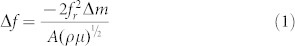
where *f_r_* is the resonant frequency, *μ* and *ρ* are the shear modulus and the density of the substrate, respectively, *A* is the sensing area. Figure 4b shows the corresponding surface mass density (*Δρ_s_* = *Δm/A*) change of the adsorbed water on the surface at various humidity for sensors D5-D10, indicating that the surface mass density increases non-linearly with the linear increase in humidity. Since the mass increase is associated with the number of water molecules, it actually reflects the nonlinear increase of water molecules adsorbed when the humidity increases. The sensor with thicker GO layer has higher capability to adsorb more water molecules, hence larger frequency shift. Note that we normalize the surface mass per unit area to eliminate the GO coverage difference from sample to sample. The increased frequency shift, hence the sensitivity, is attributed to the improved hydrophilicity and surface area of the active GO layers used. Assuming the hydrophilicity for ZnO and GO is similar, the increased surface mass density can be considered as a result of the increased sensing area, that can be calculated with respect to that of sample D1 with clean surface (see details in Figure S4 in SI). The equivalent sensing surface increases by a factor of 22, 76, 195 and 240, respectively, for samples D5, D6, D7 and D8 with different GO nanoflakes thicknesses. The results clearly demonstrated that the effective surface area of the sensing layer can be increased remarkably by using thicker and porous nanomaterials.

The frequency responses to humidity are approximately exponential as shown in Figure S5 (see SI) for D7 and D10 (same GO layer but different *f_r_*) as an example. They can be described by the following relation, 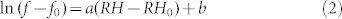
where *f_0_* is the initial resonant frequency, *RH_0_* is the reference relative humidity, *a* and *b* are two coefficient constants. The relationship has excellent linearity (0.9763<R^2^<0.9774) for both the sensors. The sensitivity of a humidity sensor is defined as^29^

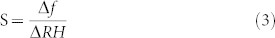
where *Δf* and *ΔRH* are the frequency shift and humidity change, respectively. From Eq.(3), the humidity sensitivity of all the sensors can be calculated with the results summarized in Table 1. The sensitivities of sensors D9-D11 with *f_r_*~225 MHz are 70–90% higher than those of sensors D6-D9 with similar GO layers but different resonant frequency, *f_r_*~140, showing that the resonant frequency has a significant effect on sensitivity of the SAW sensors. The sensitivity of the sensors increases as the resonant frequency increases.

As the sensor with a GO thickness of 100–130 nm has high sensitivity and fast response, it has been investigated in detail. Figure 4c shows the detailed frequency shift as a function of humidity of sample D10. When the relative humidity is changed from 10%RH to 85%RH with a 10%RH interval, the frequency shift again shows nonlinear characteristics. Figure 4d is the summaries of *Δf* and insertion loss vs. *RH* when the humidity is changed from 10%RH to 85%RH and back to the initial value, showing little hysteresis to the humidity change. The *f_r_* shifts down, while the insertion loss increases with humidity. Both the frequency and insertion loss show fast response when the humidity is changed in a stepwise fashion. This indicates that the residual moisture in GO layer is very limited, and is not detrimental to the accurate measurement of humidity, even when fast response is necessary.

In general, adsorption of moisture decreases the surface complex impedance. This, in turn, may change both the acoustic velocity and attenuation of a SAW device^49^. It is well known that there exists a surface conductivity window in which the velocity of the acoustic wave and attenuation are strongly correlated to the surface conductivity^49^,^50^. However, if the conductivity-induced frequency change is not in the window, it will have little effect on the resonance and attenuation of the SAW device. We noted that the working frequencies of our SAW devices (*f_r_*~140 and 225 MHz) are far away from the ionic and dipole relaxation frequency of water molecules. Therefore, the impedance (conductivity) change induced by water molecules at SAW resonant frequencies is expected to be negligible. A detailed discussion about the surface conductivity window is presented in SI (Figure S3).

**Figure 5:**
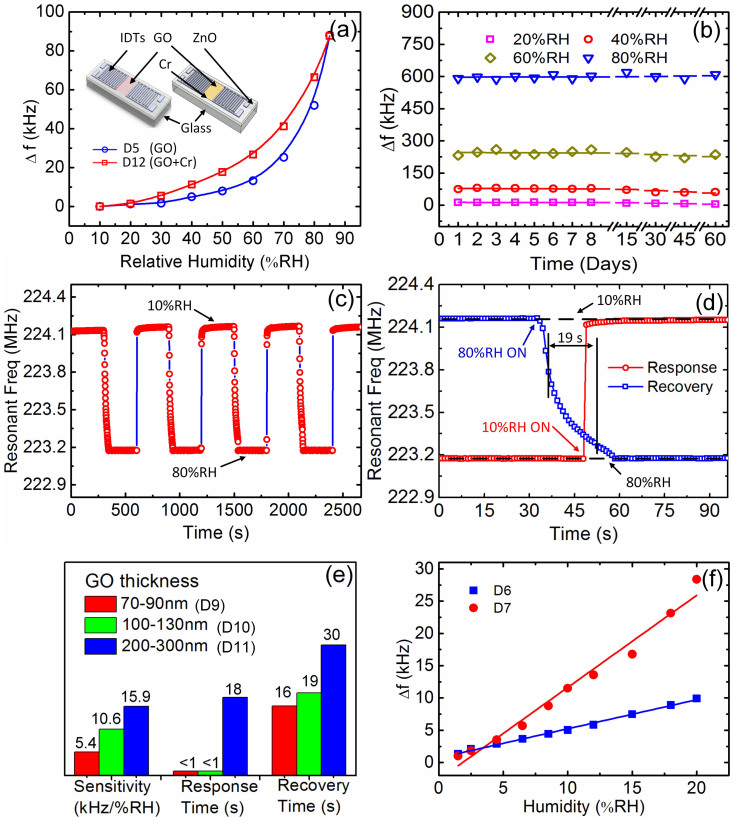
(a) Comparison of the frequency shift as a function of humidity for D5 and D12. The inset shows schematics of samples D5 and D12, respectively. (b) Stability of the performance of the SAW sensor (D10) under various relative humidity conditions, showing stable performance for over 60 days. (c) Responding and repeated characteristics of the sensor (D10) when the relative humidity is changed rapidly between 10%RH and 80%RH, showing the fast rise time < 1 sec and fall time of ~19 sec from 80%RH to 10%RH in (d). (e) The tested samples D9, D10 and D11 (with *f_r_*~225 MHz) show that the sensitivity of the sensors as well as the rise and fall times increases when the thickness of GO layer increases. (f) Frequency shift of the sensors with drop-casting GO layer at low humidity range from 0.5%RH to 20%RH (*Y* = a + b*X*, for D6: *Y* = 0.449 + 0.731*X*, *R^2^* = 0.9959; for D7: *Y* = 1.427-2.634*X*, *R^2^* = 0.9770, *R^2^* exhibit the linearity).

To further determine whether the resonant frequency shift is caused by the conductivity change or not, we designed an experiment with samples D5 and D12 (group C samples, see the inset of Figure 5a), which have the identical device structure and similar drop-cast GO layer between the IDTs. However, D12 has an extra 30 nm Cr layer deposited by e-beam evaporation (NEXDEP, Angstrom Engineering Inc.) under the GO layer. Since the Cr layer has very high electrical conductivity (*σ_sh_* ~ 0.2 Ω^−1^), it will screen off any minor surface conductivity change of the GO layer induced by adsorbed water. Therefore, the structure of D12 can eliminate the effect caused by the surface conductivity variation, and a smaller frequency shift is expected for D12 than that of D5 if there is a water-induced conductivity change in the narrow window. Figure 5a shows the frequency shift of the two samples as a function of humidity. D5 shows even less frequency shift compared to that of D12 over the most humidity range, confirming that the frequency shift of D5 is caused mainly by mass loading, rather than the conductivity variation. The slight difference in the response of the middle humidity change for D5 and D12 may be caused by the difference of the GO layers as the drop-casting may not produce identical thickness of GO layers or by the additional water adsorption by exposed Cr layer surface to moisture.

### Stability and response speed

The stability of performance is very important for any type of sensors. GO is sensitive to heat, light, and some reducing chemicals, as widely reported by a number of articles. GO is a meta-stable material whose structure and chemistry change at room temperature with a characteristic relaxation time of about one month^51^. We thus used GO material deposited on the surface of SAW devices more than a month before the sensing experiments. This is expected to significantly improve the stability of our devices. One of the approaches to further stabilize GO is to use chemical functionalization in conjunction with chemical reduction, similar to what have been proposed in Refs^39^,^52^. Here, we investigated the long-term stability of the SAW humidity sensors under a constant humidity level of 20%RH, 40%RH, 60%RH, and 80%RH, respectively. All the devices show excellent stability over a long time subjected to various humidity exposures. They do not exhibit any significant deterioration in performance. Sample D10 in Figure 5b is presented as an example where the frequency variation is less than 5% at each humidity level. The devices maintain their stable performance and sensitivity with very little drift and hysteresis for continuous sensing for over 60 days, demonstrating its excellent stability and reliability.

Sensors with fast response and recovery are desirable for applications. We thus investigated the response speed of the sensors subjected to a cyclic change of humidity level. One such example is presented in Figure 5c. The sensor shows an extremely good repeatability after tens of cyclic tests. The baseline for 80%RH remains unchanged, while that at 10%RH shifts upwards slightly, possibly due to the initial higher RH level in the environment, which desorbs from the sensing layer gradually during cyclic tests. We define the rise time as the time required for the frequency response to rise from 10% to 90% of its final value when the RH changes from 80%RH to 10%RH and the same for the fall time when RH changes from 10%RH to 80%RH. Figure 5d reveals the details of the rise and fall of resonant frequency of the sensor to humidity change, where the time interval between each data is 0.9 sec, limited by the time required for one frequency-sweeping of our network analyzer and the LabVIEW data acquisition system. The sensor exhibits ultrafast response, with a rise time less than 1 sec when the RH is decreased from 80% to 10%, table 3 shows the comparison of response speed with recent reports and a commercial product. A detailed comparison is shown in SI. Table S1 and table 4 present comparison of the sensitivity of our devices with some SAW humidity sensors reported in the literatures. The fall time of the response frequency when humidity is switched from 10%RH to 80%RH is ~19 sec, and may be dominated mostly by the permeating time of moisture to the deep GO layer. However, our device still shows potential for fast humidity sensing, especially for such a large RH change range.

The thickness of the GO layer has a significant effect on the response speed. A thicker GO layer increases both the rise and fall times as shown in Figure 5e, especially that (D11) with 200–300 nm GO layer. This is because water molecules need more time to penetrate into and escape from the GO nanoflakes layer. Figure 5e also compares the sensitivity of samples D9, D10 and D11 with the same frequency, *f_r_* ~ 225 MHz, but different GO thicknesses. The sensitivity and response times increase rapidly with the GO thickness. The details of the response curves to humidity change for these devices are shown in SI (Figure S6).

### Low humidity sensing

Although many SAW humidity sensors have been demonstrated in recent years^27^,^57^, they mostly focused on the performances at high RH levels (above 20%). Very limited effort has been made to SAW humidity sensors working at low humidity levels (<10%RH), which is of great interest of research and the industry^58^. The performance of the GO based SAW sensors with *f_r_* ~140 MHz and a GO thickness of 70–90 nm and 100–130 nm at low humidity levels (0.5%–20%RH) were further investigated (see Figure 5f). The frequency shift from 0.5%RH to 20%RH is about 9.9 kHz and 28 kHz, respectively, sufficiently large for direct circuitry readout. The sensitivity in this humidity range is about 1.448 kHz/%RH (sample D7, with GO thickness 100–130 nm) with a good linearity of R^2^>0.97, demonstrating its great potential for low humidity level sensing.

## Discussion

Generally at low humidity, the water vapor is minimal with low concentration. Water molecules are primarily physisorbed onto the available active hydrophilic groups and vacancies of the GO surface through double hydrogen bonding, that are called the first-layer physisorption of water^4^. This may explain why the induced mass change linearly depends on the surface coverage of adsorbed water at low RH. As the RH increases, multilayer physical adsorption of water molecules takes place. For the second physisorbed layer, water molecules are adsorbed through single hydrogen bonding on the hydroxyl groups. Thereafter, the water molecules become mobile and progressively behave like in the bulk liquid, which causes the exponential increase of water adsorption at high RH level, and is believed to be the reason the frequency shift increase exponentially with humidity. Sensors with flat surface would not be good enough for accurate measurement of minor frequency shift, which will also be affected severely by the noise background.

For SAW devices, the acoustic wave, hence the acoustic energy is transmitted at the surface layer of the piezoelectric substrate with a typical depth of a few micrometers^59^. If the thickness of the deposited GO film is comparable to the surface acoustic characteristic wavelength, the non-piezoelectric GO layer acts as an effective damping layer to absorb most of the acoustic wave and energy. This will deteriorate the resonance and sensitivity of the SAW devices. Our approach of using an optimized proper thickness of GO layer over the whole surface of the SAW sensors provides a very large effective sensing surface for moisture adsorption without affecting the performance of the resonators, greatly enhancing the sensitivity of the sensors at low humidity levels.

The conductivity of the GO layers is reported to be affected by the humidity^4^,^21^, different from what we observed, which may lead to the frequency shift. The difference is likely caused by several reasons. First, the conductivity change may not be located in the narrow window^49^, so that it has little effect on the response of the resonant frequency. The complex impedance induced by water adsorption in GO becomes independent of the humidity with frequency at MHz level. This is because the electric field changes so fast that the polarization of the adsorbed water cannot catch up with it, and hence the capacitance and conductivity are independent of RH at SAW resonant frequency. Second, it may also be attributed to the different treatments and the oxygen concentrations of the GO layers used, which may affect the range of conductivity changes. Further, the cleanness of the SAW surfaces and resistivity of adsorbed water may be different. Nevertheless, our results show that GO layer and coverage of the device surface are necessary for the development of high sensitivity humidity sensors.

The ionization of functional groups (especially carboxyl groups) of GO may produce protons, which may induce additional mass and change the surface conductivity. However, the change in mass on the surface is very small owing to the chemical reaction, which has little mass exchange involved, and will not change the resonant frequency. The generation of free proton carriers may partially contribute to the total change of the surface conductivity of the sensors with GO layer, which has been proven to be not the major factor to affect the resonant frequency change as discussed above. Therefore we can conclude that the possible interaction of free carrier protons via reaction of GO with water would not affect the sensing results obtained in this work.

The viscoelastic effect is another possible cause responsible for the frequency shift upon moisture absorption, and especially important for SAW humidity sensors with a polymer coating. It is difficult to separate its effect from the mass loading effect^19^,^20^. However, GO as well as graphene coating layer has a much stronger shear modulus owing to the interlock of nanoflakes than that of polymers^47^. Thus, this effect, if present, would be minor for our devices.

In summary, ZnO on glass SAW humidity sensors with GO sensitive layers have been fabricated and characterized. The sensitivity of the SAW sensors is positively correlated with GO layer thickness, resonant frequency, and the covered surface area by the GO sensitive layer. The frequency shift of the sensors is exponentially sensitive to the humidity change, and is caused by mass loading effect. The sensors with an extended coverage of a drop-casting GO layer over IDTs area have the best performance, followed by those with a GO layer in between the IDTs and those with spin-coated GO layers. The SAW sensors show very fast response to humidity change with a rise time of less than 1 sec, and the total fall time of less than 19 sec. They also show good stability at various humidity levels for up to 60 days without much deterioration. Our results demonstrate SAW devices with GO sensing layer are suitable for applications in various fields such as health and environment humidity monitoring at room temperature.

## Methods

### ZnO deposition

ZnO piezoelectric films were deposited on the glass substrate by direct-current (DC) reactive magnetron sputtering. The optimized deposition conditions have been reported previously^60^. Briefly, a 99.99% pure metallic Zn target with 100 mm diameter was used with a fixed distance of 70 mm between the target and substrate. A mixture of Ar and O_2_ with 100 and 50 sccm flow rates were employed for the deposition with the substrate temperature and the deposition pressure at 200°C and 2 Pa, respectively. The bias voltage at target was 75 V and the deposition power was 175 W. The thickness of ZnO is ~3.0 ± 0.06 μm for all the devices used in this study. After deposition, the samples were annealed at 400°C for 10 min in N_2_ atmosphere (RTP-CT100M, Premtek) to reduce the stress and defects in the films. The samples were then characterized by X-ray diffraction (XRD, Empyrean Panalytical), atomic force microscopy (AFM, SPI-3800N, Seiko Co.), and scanning electron microscopy (SEM, Hitachi S-4800) as previously reported^29^.

### SAW device fabrication and characterization

The SAW devices were fabricated on 500 μm thick Corning 2318 glass substrates with ZnO as the piezoelectric layer^60^,^61^. The interdigitated transducers (IDTs) were fabricated using standard UV-light photolithograph, followed by lift-off process with 100 nm thick aluminum (Al) layer used for the fabrication of the IDTs^60^. The SAW devices with two wavelengths of *λ* = 12 and 20 μm were fabricated to investigate the effect of resonant frequency on the sensing performance. The corresponding resonant frequencies, *f_r_*, are approximately 225 and 140 MHz, respectively. All the SAW devices have 50 pairs of IDT fingers and 10 pairs of reflecting gratings. The transmission spectrum (S_21_) and insertion loss of the sensors were investigated at various relative humidity (RH) using a network analyzer (E5071C, Agilent), which was controlled by a LabVIEW-based software through general purpose interface bus (GPIB).

### GO characteristics

Graphene oxide layers were characterized by Raman spectrocopy (inVia-Reflex, Renishaw, 532 nm laser), X-ray photoelectron spectroscopy (XPS, VG ESCALAB MARK II® system, VG instruments), X-ray diffraction (XRD, Empyrean Panalytical), and Fourier transform infrared spectroscopy (FT-IR, Nicolet 6700, Thermo Fisher scientific Inc). The thickness and morphology of the GO layers were characterized by profilometer (Alpha Step® D-100 Stylus Profiler, KLA-Tencor) and scanning electron microscopy (SEM, Hitachi S-4800).

### Humidity sensing

GO material was prepared and deposited on the surface of the SAW devices more than a month before sensing experiments. The SAW sensors were placed in a hermetic box with two through-holes for N_2_ gas with various humidity levels to pass through and two SMA (SubMiniature version A) interfaces for transmission measurements. The relative humidity in the box was controlled by changing the flow ratio of dry N_2_ to wet N_2_ into the box, while keeping the total flow rate at 500 sccm. A commercial hygrothermograph (TASI-621 TASI, based on SHT71^8^, Sensirion Inc.) was fixed at the outlet of the box to monitor the RH and temperature of the gas inside the box. All the experiments were conducted at 25 ± 2°C.

## Author Contributions

W.X., M.H. deposited ZnO and Al thin films, fabricated SAW devices; Z.X. provided the GO samples; W.X., N.M. conducted the characterization of the GO materials. W.X., X.H., W.W. and J.C. set up the humidity sensing experiment and assisted the sensing experiments. W.X. and T.S. measured all the sensors. Y.X., T.H. and J.K.L. supervised the project, analyzed the results and modified and revised the paper. All authors contributed to the discussions.

## Supplementary Material

Supplementary Information Supplementary Information

## Figures and Tables

**Table 1:** Summary of the sensors with different GO layers

Sample	*f_r_* (MHz)	GO Sensing Area(m^2^)	Sensitivity(kHz/5%RH)	Surface Treatment
D1	~140	No	7.21	Clean surface without GO
D2	~140	1.84e-5	6.25	Spin-coating GO on the whole surface (GO 0.046 mg/ml)
D3	~140	1.84e-5	12.51	Spin-coating GO on the whole surface (GO 0.184 mg/ml)
D4	~140	1.84e-5	10.29	Spin-coating GO on the whole surface (GO 0.460 mg/ml)
D5	~140	3.7e-6	36.14	Drop-casting in between IDTs (GO thickness 70–90 nm)
D6	~140	1.84e-5	55.00	Drop-casting on the whole surface (GO thickness about 70–90 nm)
D7	~140	1.84e-5	58.12	Drop-casting on the whole surface (GO thickness about 100–130 nm)
D8	~140	1.84e-5	97.29	Drop-casting on the whole surface (GO thickness about 200–300 nm)
D9	~225	9.16e-6	81.03	Drop-casting on the whole surface (GO thickness about 70–90 nm)
D10	~225	9.16e-6	152.58	Drop-casting on the whole surface (GO thickness about 100–130 nm)
D11	~225	9.16e-6	265.18	Drop-casting on the whole surface (GO thickness about 200–300 nm)
D12	~140	3.7e-6	21.29	Drop-casting in between IDTs (GO thickness 70–90nm) with a 30 nm Cr underneath the GO layer

**Table 2:** The sorted groups of the sensors

Group	Samples	Description
Group A	D1–D8	Sensors with same *f_r_* but different GO layers
Group B	D7, D10	Sensors with different *f_r_* but same GO layers
Group C	D5,D12	Sensors with or without a Cr layer

**Table 3:** The response speed comparison of humidity sensors

Sensor type	Sensing material	Fabrication method	Hum. & Temp. range (RH, ^o^C)	Recovery & response time	Ref.
SAW	GO	Spin and drop-casting	0.5%–85%, 20–50	<1s, 19 s	This work
Capacitance	GO	Drop-casting	15%–95%, 25	41 s, 10.5 s	4
QCM-type	GO	Spin-coating	6.4%–93.5%, 25	12 s, 18 s	33
Resistance	PDDA/RGO	LbL self-assembly	11%–97%, 25	94 s, 108 s	53
SAW	GO	Droplet by SAW atomizer	5%–100%, 25	n/a	31
Resistance	nanofibre	Drop-casting	5%–80%, 10–50	8 ms, 24 ms	54
Capacitance	polymer	n/a	0%–100%, -40–125	8 s, n/a	8
Impedance	rGO	Spin and drop coating	35%–80%, 20–40	30 ms, 30 ms	23

**Table 4:** Comparison of sensitivity of SAW-based humidity sensors

*f_r_* (MHz)	Substrate	Sensing material	Sensitivity(kHz/5%RH)	Reference
~225	ZnO	GO	~265	This work
~150	AlN	ZnO nanorods	~60	55
~132	ZnO/PI	ZnO	~17	29
~120	LiNbO3	Silver Nanostructures	~130	30
~500	ST-quartz	MWCNTs/Nafion nanofibers	~2000	56
433	ST-quartz	Silicon-containing polymer	~2	27
