# Supplementary material for: Fast Response and High Sensitivity ZnO/glass Surface Acoustic Wave Humidity Sensors Using Graphene Oxide Sensing Layer
Source: Sci Rep. 2014 Nov 26;4:7206. doi: 10.1038/srep07206 (PMC4244623; doi:10.1038/srep07206)
Supplement: Supplementary Information [file srep07206-s1.pdf]

Supplementary information

Fast Response and High Sensitivity ZnO/glass Surface Acoustic Wave Humidity Sensors Using Graphene Oxide Sensing Layer

Weipeng Xuan[1], Mei He[1], Nan Meng[1], Xingli He[1], Wenbo Wang[1], Jinkai Chen[1], Tianjin Shi[1], Tawfique Hasan[2], Zhen Xu[3], Yang Xu[1*] and J. K. Luo[1,4*]

1. Dept. of Info. Sci. & Electric. Eng., Zhejiang University and Cyrus Tang Center for Sensor Materials and Applications, 38 Zheda Road, Hangzhou 310027, P.R. China.

2. Cambridge Graphene Centre, University of Cambridge, Cambridge CB3 0FA, United Kingdom

3. MOE Key Lab. of Macromolecul. Synth. & Funct., Dept. of Polym. Sci. & Eng., Zhejiang University, 38 Zheda Road, Hangzhou 310027, P. R. China.

4. Inst. of Renew. Energ. & Environ. Technol., University of Bolton, Deane Road, Bolton BL3 5AB, United Kingdom

*Corresponding authors. E-mails: yangxu-isee@zju.edu.cn; jackluo@zju.edu.cn

1. Thickness of GO layers

GO dispersions with different concentrations were used to produce the GO sensitive layers with various thicknesses on the surface of SAW devices by drop-casting. The thickness of the GO layers was measured by profilometer. Figure S1 shows the thickness variation for the drop-casting samples with different GO concentrations. The surface of the GO layers by drop-casting is very rough, and the average thickness of the GO layers can be estimated from the measurements to be approximately 70-90 nm, 100-130 nm, and 200-300 nm for the samples drop-cast with 0.046, 0.186 and 0.460mg/ml GO concentration, respectively. We

found that a GO layer thicker than 500-600 nm can render the device inoperable with drastically deteriorated resonant spectrum[1] and diminished resonance as shown in Figure S2. The GO layer thickness is thus limited to 200-300 nm for all the sensors used for this work.

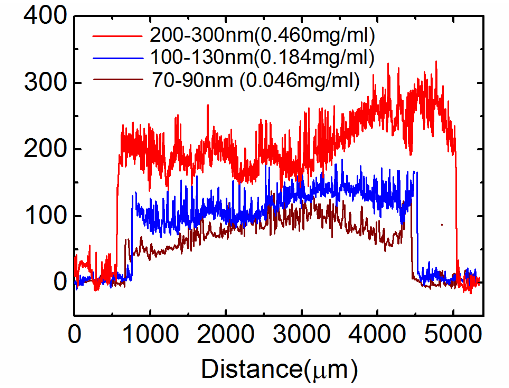

Figure S1. The thickness profiles of GO measured by profilometer.

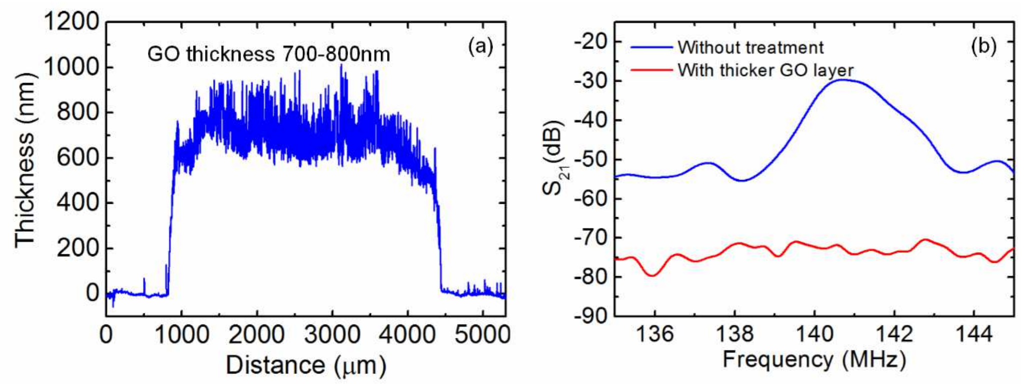

Figure S2. The thickness of GO layer (a) and (b) the transmission spectrum of the SAW device before and after a 700-800nm GO layer deposited.

2. The effect of surface conductivity on the resonance

Moisture adsorbed on the surface induces not only a mass loading to a device, but may also change the conductivity of the surface layer.[2] Generally, the change of surface conductivity will shift both the acoustic resonant frequency and attenuation of the acousto-electric interaction, *i.e.* the transmission signal amplitude. It is known that within a narrow conductivity window, they are strongly correlated indicated by the following formulas[3, 4]

$$\frac{\Delta v}{v_0} = -\frac{K^2}{2}\frac{\sigma_{sh}^2}{\sigma_{sh}^2 + v_0^2 C_s^2} \quad \text{(EQU-S1)}$$

$$\frac{\alpha}{k} = -\frac{K^2}{2}\frac{v_0 C_s \sigma_{sh}}{\sigma_{sh}^2 + v_0^2 C_s^2} \quad \text{(EQU-S2)}$$

where K^2 is the electromechanical coupling coefficient, σ_{sh} is the surface conductivity of the sensitive film, C_S is the capacitance per unit length of the surface, α is the attenuation, k is the wave number, and α/k is the acoustic-electric interaction attenuation per wave number. Figure S3 shows the calculated acoustic velocity shift and attenuation as a function of surface conductivity change,[3, 5, 6] clearly demonstrating the existence of a narrow surface conductivity window in which the velocity and attenuation of the acoustic waves are strongly correlated to the surface conductivity.[3, 4] Furthermore, the SAW devices used in this work have resonant frequency of about 140 and 225 MHz, far away from the ionic and dipole relaxation frequency of water molecules, where it is typically in the range of Hz to hundreds of kHz.[7] We believe this is the main reason that the humidity-induced conductivity change has little effect on the resonant frequency of our SAW humidity sensors.

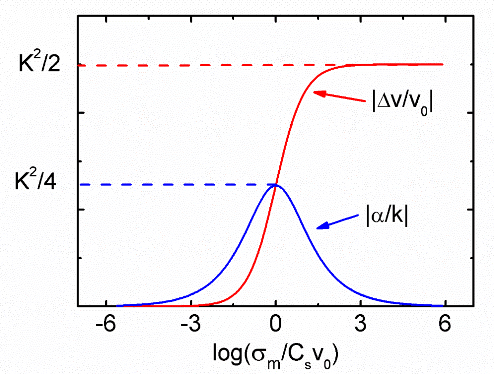

Figure S3. Acoustic velocity shift and acousto-electric attenuation as a function of surface conductivity of a thin film on the ZnO piezoelectric layer. The electromechanical coupling coefficient K^2 is assumed to be constant in these calculations.

3. The mass loading effect

Assume the hydrophilicity for ZnO and GO is similar, then the increased surface mass density obtained can be treated as the increased sensing areas. For comparison, we have plotted the surface mass density ratio of D5-D10 with respect to that of sample D1 with clean surface in Figure S4. D5 with the GO in between the IDTs has the same sensing area as that of D1. The use of GO layer equivalently increases the sensing surface by a factor of 22. For samples D6-D10 with different thicknesses of drop-casting GO layers, the equivalent sensing surface increases by a factor of 76, 195, 240, 74 and 140 respectively, at humidity 60%RH, with the various GO thickness. The results clearly demonstrated that the effective surface area of sensing layer can be increased remarkably by using thicker and porous nanomaterials.

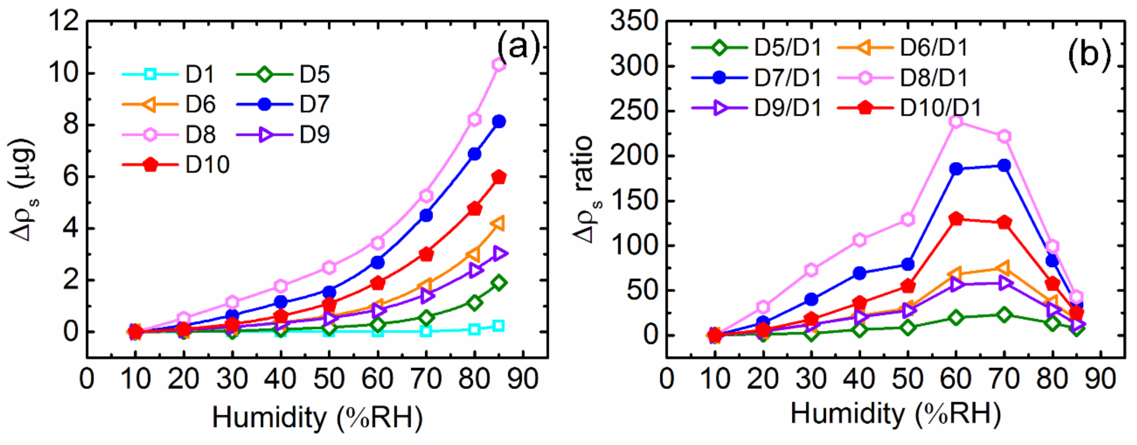

Figure S4. The adsorbed surface mass as a function of relative humidity (a) and (b) the surface mass ratios of D5/D1, D6/D1, D7/D1, D8/D1, D9/D1, and D10/D1.

4. Frequency and GO thickness effects on response to humidity

The resonant frequency has a significant effect on sensitivity of the SAW sensors. Figure S5a shows the frequency shift as a function of humidity for the two sensors with different frequencies but a similar GO layer (Group B, GO thickness 100-130 nm). When the relative humidity is changed from 10%RH to 85%RH in a 10%RH interval, the frequency shift of the two devices show nonlinear characteristics as observed in Figure S5a. After re-plotting them

in Figure S5b, it is clear that the frequency responses to humidity are approximately exponential with excellent linearity (0.9763<R^2<0.9774).

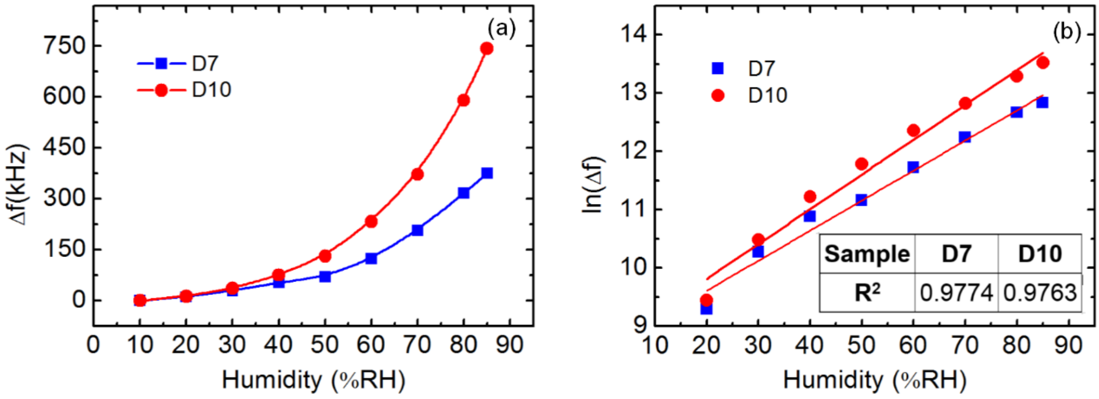

Figure S5. The frequency shift as a function of humidity for SAW sensors (group B) with different resonant frequencies in linear plot (a) and in semi-log plot (b). All the devices have a drop-casting GO layer.

The thickness of the GO layer has a significant effect on the response speed. A thicker GO layer increases both the rise time and fall time as shown in Figure S6, especially that with 200-300 nm GO layer. This is because water molecules need more time to penetrate into and escape from the GO layer. The sensitivity and response times increase rapidly with the GO thickness. Figure S6 shows the frequency response of the sample D9, D10 and D11 with the same f_r~225 MHz but different GO thicknesses between the humidity 80%RH and 10%RH, clearly showing the rapid increase of the response time with the GO thickness.

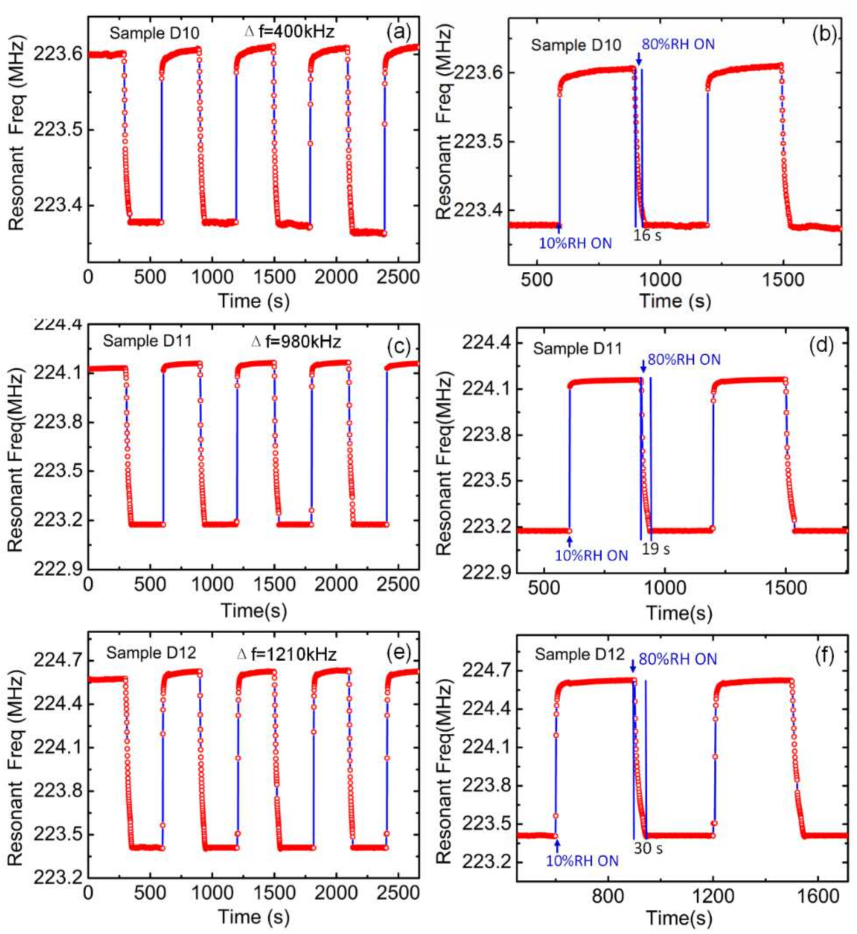

Figure S6. The response of the sensors subjected to a cyclic change of humidity level, showing the fast response from 80%RH to 10%RH. The sensors also have an extremely good repeatability after tens of cyclic tests and the zoom-in images show the detailed response of the sensors.

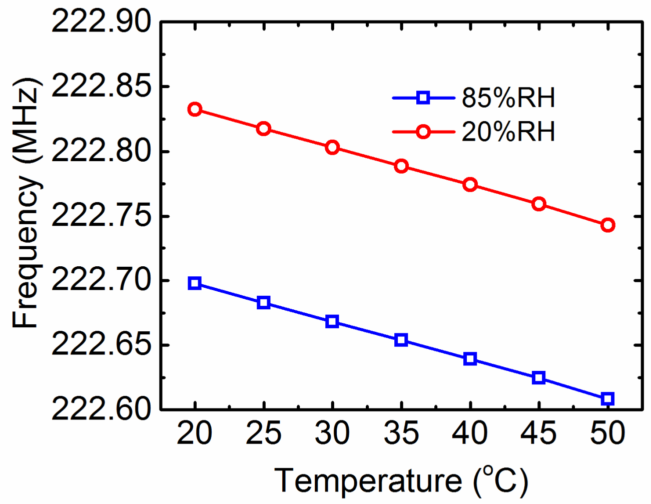

Figure S7. Temperature dependence of the SAW device at 20%RH and 85% RH.

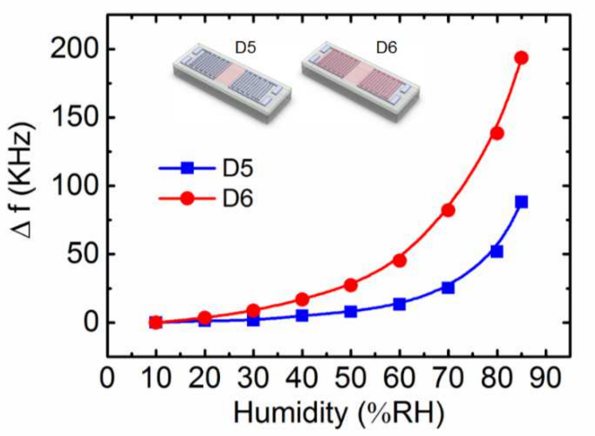

Figure S8. The sensitivity comparison of Samples D5 and D6 with the same thickness GO film but different GO sensing area.

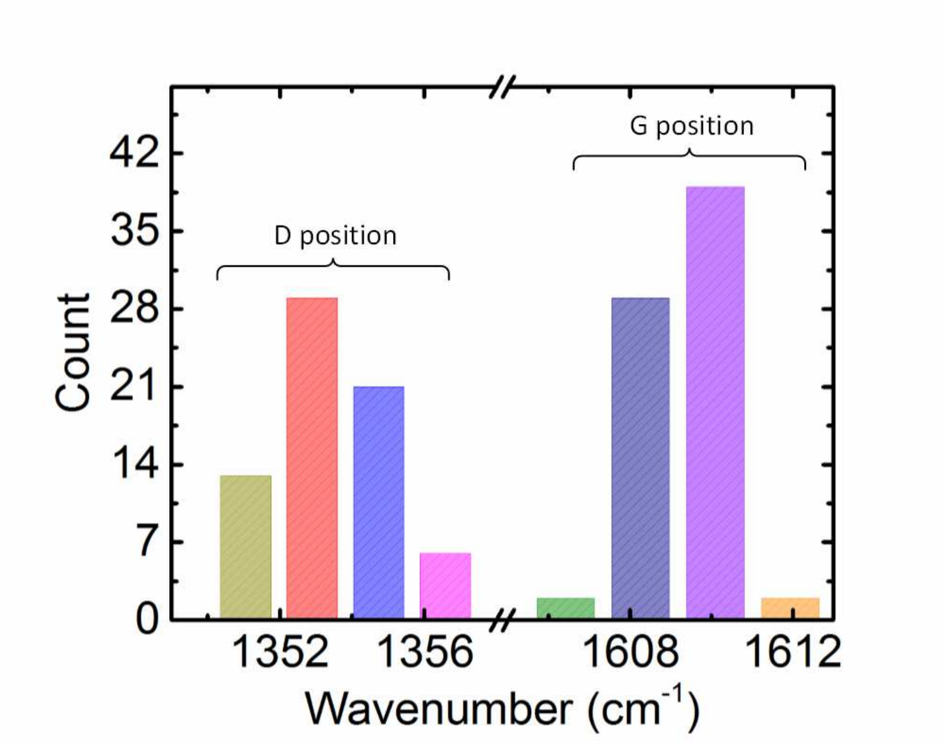

Figure S9. Raman characterization of GO at multiple random points in a large area shows a good uniform distribution of GO on our SAW surfaces. The excitation laser wavelength is 532 nm.

Table S1. Comparision of high-performance inorganic humidity sensors

Sensor type	Sensing material	Humidity & Temperature Range	Recovery & response time	Sensitivity	Reference
SAW	GO	0.5%-85%RH 20 ºC -50 ºC	1s, 19 s	~265 (kHz/5%RH)(at 85%RH) f_0=~225MHZ	This work
SAW	MWCNTs/Nafion nanofibers	10%-80%RH @RT	3s, 3s	~2000(kHz/5%RH)(at 85%RH) f_0=~500MHZ	S1
SAW	Nanostructure SiO2	0-90% @25 ºC	n/a	33kHz/%RH f_0=120MHz	S2
SAW	Polyaniline nanofiber	5-90% @25 ºC	n/a	4.58kHz/%RH f_0=145MHz	S3
SAW	ZnO nanorods	10%-90% @25 ºC	n/a	10kHz/%RH	S4
SAW	Silicon-containing polyelectrolyte	11%-75% @RT	n/a	1kHz/%RH f_0=433MHz	S5
SAW	Nanocrystalline ZnO	10%-90% @RT	n/a	2.25kHz/%RH f_0=125MHz	S6
SAW	PVA and PVP	2%-95% @RT	1.5s,2.5	n/a	S7
R	VS_2 ultrathin nanosheets	0%-100%RH @ 25 ºC	30s, 12s	30	S8
R	V_2O_5 nanotubes	11% -97%RH @25 ºC	8s, 20s	23	S9
R	SnO_2 NW	5%-85%RH @30 ºC	120s, 20s	35 @ 85%RH	S10
R	Al doped n-ZnS	30%-90%RH @RT	95s, 209s	200 @90%RH	S11
R	CeO_2 nanowires	15%-97%RH @RT	3s,3s	85@97%RH	S12
R	Si thin film	20%-92%RH @20 ºC	0.2s	10000	S13
R	ZnO nanorods	12%-97%RH @RT	3s,20s	183	S14
R	VO_2 nanostructures	11%-97%RH @25 ºC	5s,2s	50@97%RH	S15
R	$MgFe_2O_4$	10%-90%RH	4s,6s	20888	S16

	thin films	@25 ºC			
R	ZnO thin film	6.3%-84%RH @30 ºC	3s,12s	42678	S17
R	$BaTiO_3$ nanofiber	11%-95%RH @RT	20s,3s	120	S18
C	Amorphous Al_2O_3 nanotubes	11%-95%RH @RT	10s,20s	10000	S19
C	TiO_2	1%-95%RH @RT	0.22s, 0.4s	1000	S20
C	TiO_2 thin film	0%-90%RH @RT	0.05s, 3s	19nF/%RH	S21
C	Amorphous TiO_2	2%-92%RH @RT	0.27s, 0.29s	1000 @ 92%RH	S22
C	TiO_2 nanofilms	2%-95%RH @RT	0.06s, 1.44s	1000	S23
I	LiCl Doped TiO_2 nanofibers	11%-95%RH @25 ºC	3s, 7s	6600	S24
I	Porous TiO_2	11%-95%RH @RT	5s, 8s	1.19 MΩ/%RH	S25
I	$Na_2TiO_3O_7$ nanowires	11%-95%RH @25 ºC	4s,5s	10000	S26
I	Graphene oxide	35%-85%RH 20 ºC -40 ºC	0.03s, 0.03s	n/a	S27

* R: Resistive, C: Capacitive, I: Impedance; @RT: Room temperature

S1.Lei, S.et al. *Nanotechnol.* 2011, *22*, 265504.

S2. Kwan, J.K.et al. *Sens. Actuators, B* 2012, *173*, 164–168.

S3. Wu, T.-T.et al. *J. Phys. D: Appl. Phys*. 2008, *41*, 085101.

S4. Hong, H.S.et al. *Sens. Actuators, B* 2012, *171*, 1283–1287.

S5. Lei, S.et al. *Sens. Actuators, A* 2011, *167*, 23–236.

S6. Hong, H.-S.et al. *Sens. Actuators, B* 2010, *148*, 347–352.

S7. Buvailo, A.et al. *Sens. Actuators, B* 2011, *156*, 444–449.

S8. Feng et al. *Adv. Mater*. 2012, *24*, 1969–1974.

S9. Yin et al. *J. Mater. Chem*. 2012, *22*, 5013–5019.

S10. Kuang et al. *J. Am. Chem. Soc*. 2007, *129*, 19, 2007– 6071.

S11. Luo et al. *J. Mater. Chem*. 2012, *22*, 6856–6861.

S12. Wang et al. *Nanotechnology* 2007, *18,* 145503.

S13. Kalkan et al. *IEEE Electron Device Letters*, 2004, *25*, 8*,* 526.

S14. Luo et al. *Applied Surface Science* 2005, *242,* 212–217.

S15. Zhu et al. *Electroanalysis* 2011, *23*, 7, 1752 – 1758.

S16. Kotnala et al. *Thin Solid Films* 2011, *519,* 6135–6139.

S17. Kannan et al. *Sensors and Actuators A* 2010, *164,* 8–14.

S18. Wang et al. *Sensors and Actuators* B 2011, *153,* 460–464.

S19. Lei et al. *J. Mater. Chem*. 2011, *21*, 1907–1912.

S20. Steele et al.*IEEE Sensors Journal* 2007, *7*, 6, 955.

S21. Kupsta et al. *IEEE Sensors Journal* 2009, *9*, 1979.

S22. Steele et al. *IEEE Sensors Journal*, 2008, *8*, 1422.

S23. Steele et al. *Sensors and Actuators B* 2009, *140*,610–615.

S24. Wei et al. *J. Am. Chem. Soc*. 2008, *130*, 5036–5037.

S25. Zhang et al. *Nanotechnology* 2011, *22,* 275502.

S26. Zou et al. *Sensors and Actuators B* 2008, *135,* 317–321

S27. Borini et al. *ACS Nano* 2013, *7*, 11166-11173.

References

[1] Huang, F.C., Chen, Y.Y., Wu, T.T., A room temperature surface acoustic wave hydrogen sensor with Pt coated ZnO nanorods, *Nanotechnol.* **20**,065501 (2009).

[2] Lin, Q., Li, Y., Yang, M., Highly sensitive and ultrafast response surface acoustic wave humidity sensor based on electrospun polyaniline/poly(vinyl butyral) nanofibers, *Anal. Chim. Acta* **748**,73-80 (2012).

[3] Ricco, A.J., Martin, S.J., Zipperian, T.E., Surface Acoustic-Wave Gas Sensor Based on Film Conductivity Changes, *Sens. Actuators* **8**,319-333 (1985).

[4] Fan, L., et al., Influence of surface conductivity on sensitivity of acoustic wave gas sensors based on multilayered structures, *IEEE Trans. Ultrason. Ferroelectr. Freq. Control* **58**,451-460 (2011).

[5] Takeuchi, M., Yamada, H., Yoshino, Y., Makino, T., Arai, S., Effective electromechanical coupling coefficient

(kt2) for fundamental mode of thickness extensional mode thin film bulk acoustic wave resonator fabricated by ZnO thin film, *Vacuum* **66**,463-466 (2002).
[6] Mahmood, F.S., Gould, R.D., Hassan, A.K., Salih, H.M., DC properties of ZnO thin films prepared by r.f. magnetron sputtering, *Thin Solid Films* **270**,376-379 (1995).
[7] Bi, H., et al., Ultrahigh humidity sensitivity of graphene oxide, *Sci. Rep.* **3**,2714 (2013).
